# The Retrospective Analysis of Posterior Short-Segment Pedicle Instrumentation without Fusion for Thoracolumbar Burst Fracture with Neurological Deficit

**DOI:** 10.1155/2014/457634

**Published:** 2014-03-02

**Authors:** Zhouming Deng, Hui Zou, Lin Cai, Ansong Ping, Yongzhi Wang, Qiyong Ai

**Affiliations:** ^1^Department of Orthopaedic, Zhongnan Hospital of Wuhan University, No. 169 Donghu Road, Wuhan, Hubei Province 430071, China; ^2^Department of Orthopaedic, Central Hospital of Huanggang City, Huanggang, China; ^3^Department of Urology, Zhongnan Hospital of Wuhan University, Wuhan, China

## Abstract

This study aims to investigate the efficacy of posterior short-segment pedicle instrumentation without fusion in curing thoracolumbar burst fracture. All of the 53 patients were treated with short-segment pedicle instrumentation and laminectomy without fusion, and the restoration of retropulsed bone fragments was conducted by a novel custom-designed repositor (RRBF). The mean operation time and blood loss during surgery were analyzed; the radiological index and neurological status were compared before and after the operation. The mean operation time was 93 min (range: 62–110 min) and the mean intraoperative blood loss was 452 mL in all cases. The average canal encroachment was 50.04% and 10.92% prior to the surgery and at last followup, respectively (*P* < 0.01). The preoperative kyphotic angle was 17.2 degree (±6.87 degrees), whereas it decreased to 8.42 degree (±4.99 degrees) at last followup (*P* < 0.01). Besides, the mean vertebral body height increased from 40.15% (±9.40%) before surgery to 72.34% (±12.32%) at last followup (*P* < 0.01). 45 patients showed 1-2 grades improvement in Frankel's scale at last followup. This technique allows for satisfactory canal clearance and restoration of vertebral body height and kyphotic angle, and it may promote the recovery of neurological function. However, further research is still necessary to confirm the efficacy of this treatment.

## 1. Introduction

Owing to the fulcrum of increased motion at the T12-L1 junction, approximately 90% of spine fractures are located in the thoracolumbar region, and the burst fractures account for nearly 10–20% of spine injuries [1]. The definition of “burst fracture” was firstly presented by Holdsworth in 1963 [2]. These injuries mainly occur in younger patients associated with falls or motor vehicle accidents. Based on the 3 columns theory of Denis [3], thoracolumbar burst fracture often leads to compression fracture of anterior and middle vertebral columns and an associated kyphotic deformity, and such compression fracture can cause the retropulsion of bone fragment into the spinal canal. Even it is a common fracture, especially in the developed countries, the optimal managements are still controversial [4, 5]. The controversies about this topic mainly focus on whether operation for such fracture is worthy and which surgical approach, anterior or posterior, is more effective in the treatment of thoracolumbar burst fracture. Although the nonoperative treatment was considered as a choice for thoracolumbar burst fracture without neurologic deficit and stable fracture, it is believed to have shortcomings such as persistently low back pain and neurologic deterioration owing to incorrect alignment. Besides, this treatment involves prolonged bed rest, which may increase the risk of complication (thromboembolism, pulmonary complication, and so on). In contrast, although the surgical therapy is physically invasive for patients, it can offer immediate spinal stability, early mobilization, decompression of neural elements, and more reliable correction of kyphosis, as well as vertebral and canal dimensions [6, 7].

The aims of surgical treatment for thoracolumbar burst fracture are as follows: (1) promoting neurological recovery by decompression of spinal canal and nerve roots, (2) obtaining rigid fixation to prevent delayed neural injuries, (3) restoration and maintenance of anatomic alignment, (4) relieving pain and facilitating early rehabilitation, and (5) limiting the number of instrumented centrums [4, 7, 8]. With the advantages of 3-column fixation characteristics, combination of forces (distraction, compression, and rotation), less invasive compared with anterior approach, safety exploration, and less technical demanding, the posterior pedicle screw fixation is most frequently used today [9–11].

Compared with long-segment posterior fixation (LSPF), the short-segment posterior fixation (SSPF) is easy to implement, offering the advantage of preserving spinal motion segments, shorter operative time, and less blood loss. With the improvement of rigidity and stiffness of pedicle screw-based posterior spinal instrumentation systems, the short-segment has become more reliable [12]. Even the spinal canal remodeling is shown to occur regardless of operative or nonoperative treatment, the “surgical clearance” can improve the neurological outcome partially [13]. Several studies have found that the restoration of normal canal dimensions may be associated with the recovery of neurological function for patients with partial deficits [14–16]. In other words, reduction of deformity and retropulsed bone fragment in the canal may play an important role in obtaining optimal surgical outcome. There are no specific tools for restoring retropulsed bone fragment. Based on this, we designed a novel tool, which was named as repositor for retropulsed bone fragments (RRBF). The details of RRBF were described in this study, and the long-term results of thoracolumbar burst fracture treated with SSPF combined RRBF were also showed.

## 2. Methods and Materials

The inclusion criteria of this study were a single-level thoracolumbar burst fracture (T11-L1) with neurological deficit and the intraspinal bone fragments confirmed by CT. The exclusion criteria were as follows: patients with pathological fractures, multiple level fracture, polytraumatized patients, and a history of spine surgery and preexisting neurological deficit. From July 2009 to Aug 2011, 53 patients were in accordance with the inclusive criteria. Among the 53 patients, there are 19 females and 34 males, ranging from 18 to 63 years (average 34.5 years). The major mechanism of injury was fall and traffic accidents. According to the classification described by Magerl et al. [17], 17 cases belonged to subtype A 3.1, 32 cases belonged to subtype A 3.2, and the rest 4 cases belonged to subtype A 3.3. The neurological deficits were assessed by the operating surgeons on the basis of American Spinal Injury Association's Modified Frankel's grading of traumatic paraplegia [18]. The involved levels were T11 in 13 patients, T12 in 19 patients, and L1 in 21 patients.

### 2.1. Radiological Evaluation

**Figure 1:**
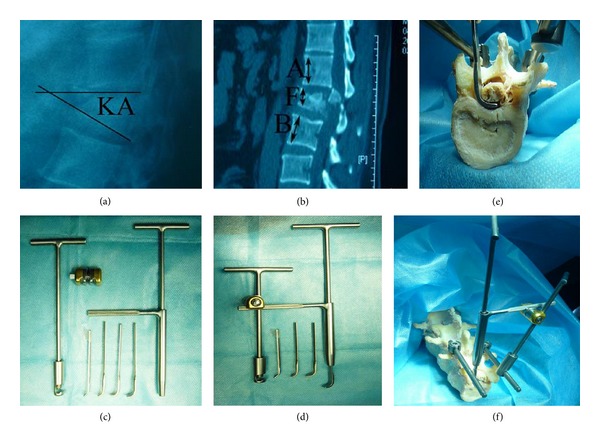
(a)-(b) The diagram of kyphotic deformity and vertebral body height calculated in current study. Vertebral body height = 2*F*/(*A* + *B*)∗100. (c)-(d) The physical map of a novel custom-designed repositor (RRBF). (e)-(f) The usage and preclinical application of RRBF.

Anteroposterior and lateral radiographs, CT, and magnetic resonance imaging (MRI) of the thoracolumbar region were performed in all patients on admission. The segmental kyphotic angle of fractured vertebra was measured as the angle between the upper margin of the vertebral body and the lower margin of the vertebral body. The vertebral body height was calculated by the formula adopted by Haas et al. [19]. The percentage of canal compromise was measured according to the formula *α* = (1 − *x*/*y*)∗100, which was described by Hashimoto et al. [20] (Figures 1(a)-1(b)). The *x* in this formula was defined as the least midsagittal of the spinal cord of the fractured vertebra, and the *y* was calculated by the average of midsagittal canal diameter of the adjacent vertebra, which was considered as the normal midsagittal canal of the injured vertebral body.

### 2.2. Surgical Technique

All patients were operated in prone position, with a midline skin incision extending above and below 1 or 2 levels of the fractured levels. The paraspinal musculature was dissected bluntly. Aiming to achieve temporary stability of the spine, the short-segment pedicle instrumentation was fixed before posterior decompression was implemented. The fixation was achieved by inserting four screws into the pedicles of the adjacent vertebra above and below the injured level, and then rods with appropriate length were inserted and connected to the screws. Some reduction techniques such as distraction of anterior and posterior column and in situ bending of the rods were used, and then the screws were fixed tightly. After conventional laminectomy, the reduction of the retropulsed bone fragments was conducted by using a novel custom-designed instrument.

### 2.3. A Novel Repositor for Retropulsed Bone Fragments

This novel custom-designed repositor for retropulsed bone fragments (NO. ZL201020673890.X, CN PAT) was designed by the corresponding author and manufactured by Dragonbio (Hubei, China). It consisted of two parts, the supporting apparatus and reduction apparatus (Figures 1(c)–1(f)). The RRBF was connected with the rods through supporting device, and the reduction was accomplished by reduction apparatus. In brief, the application method was as follows: firstly, the supporting device was fit together with the rods of posterior instruments. According to the location of retropulsed bone fragments from preoperative CT scan, we could decide which side the supporting device is located. Secondly, we assemble the reduction apparatus, which included the reduction crank, sleeve, and compressing bar. The lower end of reduction crank was placed on the surface of retropulsed bone fragments from lateral side of dura, the upper end of reduction crank was inserted in and joint with sleeve. The reduction apparatus and supporting device was fixed via the tie rod of the sleeve. Finally, insert the compressing bar into the sleeve and concatenate the reduction crank. By twisting the compressing bar, we can achieve the restoration of retropulsed bone fragments gradually and gently. Another function of the reduction crank is that it can orientate the retropulsed bone fragments on the X-ray C-arm system.

### 2.4. Follow-Up Evaluation

All patients were immobilized by a thoracolumbar sacral orthosis brace for preventing the implant failure and promoting neurological recovery. After appropriate 4 weeks, patients with sufficient motor movement were gradually mobilized according to personal neurological status and radiological review. The restoration of vertebral height and correction of the kyphotic angle were assessed by anterioposterior and lateral film for several times which included 1 week after surgery, regular interval of 3 months, and the final follow-up examination. CT scans were also obtained to assess the percentage of postoperative spinal canal compromise using the similar formula described in radiological evaluation.

### 2.5. Statistical Analysis

The whole statistical analysis was performed with SPSS for windows version 18.0 (SPSS Inc, Chicago, USA). The paired *t*-test was used to test the restoration of vertebral height, correction of the kyphotic angle, and the canal decompression before and after operation. The association between the preoperatively neurological status and the initial canal compromise was assessed by chi-square test. *P* value <0.05 was considered statistically significant for all tests.

## 3. Results

The mean operative time in all cases was 93 min (ranging from 62 to 110 min). No patient needed blood transfusion with mean 452 mL intraoperative blood loss. There were no major complications such as death, cerebrospinal fluid leak, or epidural hematoma. As for the other complications, 2 cases suffered pneumonia which was cured by antibiotics, and one patient developed sacrococcygeal bedsore which was cured by applying vacuum-assisted closure. Patients were monitored 14–49 months (average 25.4 months) for follow-up evaluation, the implant failure (broken screws and rods) was observed after operation in two patients. The fixation was removed for all patients between 12 and 16 months after surgery.

### 3.1. Preoperative Neurological Deficit, Canal Compromise, Loss of Vertebral Body Height, and Kyphotic Angle

In this study, all patients have neurological deficit at admission. The mean spinal canal compromise in patients with complete paraplegia and incomplete paraplegia was 52.27% ± 9.73% and 50.98% ± 7.72%, respectively. The relationship between the extent of canal encroachment and the level of initial neurological status has no statistical significance. The loss of vertebral body height and kyphotic angle of all 53 patients was 40.15% and 17.21 degree, respectively. Neither the loss of vertebral body height nor kyphotic angle was significantly correlated with the initial neurological status. (Table 1).

### 3.2. The Radiological Evaluation of Fractured Vertebra

**Figure 2:**
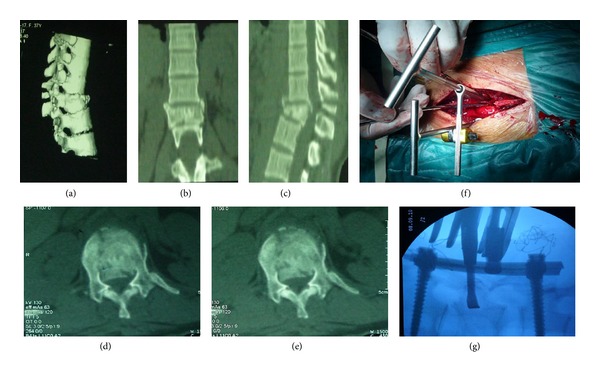
(a)–(e) The preoperative CT scan image demonstrating a significant spinal canal encroachment by retropulsed bone fragments. (f)-(g) The intraoperative application of RRBF and the fluoroscopy of restoration of fractured vertebral body.

**Figure 3:**
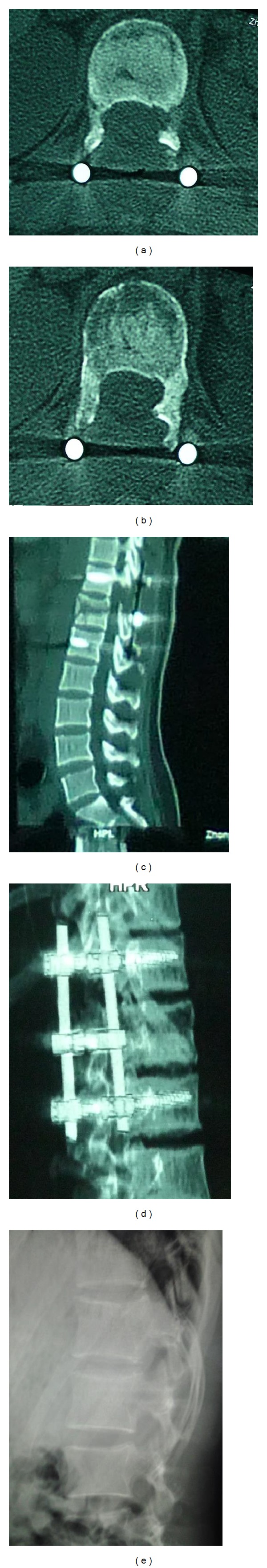
(a)–(d) The postoperative CT scan confirming satisfactory canal clearance and restoration of vertebral body height. (e) After removal of implants for one months, the lateral radiograph showed that the kyphotic angle and vertebral body height were maintained acceptably.

The average canal encroachment of retropulsed bone fragments was 50.04%, 20.04%, and 10.92% preoperatively, postoperatively, and at last followup, respectively. The difference had a statistical significance (*P* < 0.01). The preoperative kyphotic angle was 17.2 degree (±6.87 degrees), whereas it decreased to 8.42 degree (±4.99 degrees) at last followup. Such difference had statistical significance (*P* < 0.01). Besides, the mean vertebral body height was initially 40.15% (±9.40%), and then it increased to 72.34% (±12.32%) at last followup (*P* < 0.01). No patients had evidence of pseudarthrosis; typical case was shown as Figures 2 and 3.

### 3.3. The Neurological Recovery

There was no deterioration in patients' neurological status. Among the 11 patients with Frankel's grade A, 6 showed no improvement in neurological status, and the other 5 showed 1-2 grades improvement in Frankel's scale at last followup. Especially, 40 out of 42 patients with incomplete paraplegia (Grade B–D) achieved significantly neurological recovery (Table 2).

## 4. Discussion

The treatment of thoracolumbar burst fracture remains challenging and debatable. There are middle column injuries in thoracolumbar burst fracture, so a lot of surgeons think that it is an unstable spinal fracture [12]. It is generally recommended that patients who present with incomplete neurologic involvement and an unstable burst fracture need surgical intervention [21]. When surgical therapy is determined, the forthcoming problem is which approach (anterior, posterior, or combined) should be chosen. Most surgeons choose their surgical approaches based on their experience and preference, and each respective choice seems to be imperfect, and there is still no evidence confirming the advantage of any option regarding the outcome [22]. Some authors suggest that the ideal treatment is a combined approach [23], but a systematic review conducted by Oprel et al. [24] failed to identify the better one from posterior surgery and combined surgery. It is frequent that there is lack of significant difference in studies comparing different surgical approach [25]. Verlaan et al. [26] found that the discs adjacent to injured vertebra treated with posterior pedicle fixation may not progress towards significant degeneration, which was confirmed by a study of 10 years followup [27]. These results definitely make surgeons revalue surgical treatment details.

Even it is not clear that there is correlation between the neurologic recovery and the surgical approach, the pedicle screw fixation via posterior approach has been widely used for most thoracolumbar fractures owing to its 3-column fixation and satisfactory clinical outcomes [8, 28]. No matter which approach is adopted, one of the important goals of surgery is to obtain stable fixation with the least instrumented centrums. Considering of that, SSPF is frequently regarded as a valuable choice. Even there are complications such as implant failure and loss of correction in SSPF, it is easier to perform and allows more preservation of spinal motion segments. It is remarkable that the long-term complications will reduce upon the preservation of spinal motion segments using SSPF. With the development and inter- and intraobserver agreement of the load-sharing classification (LSC) [29, 30], more authors have accepted that the treatment of thoracolumbar burst fracture by SSPF will achieve good outcome on the condition of no severe anterior column defect [31–34]. The necessity of fusion is still questionable. Neither SSPF nor LSPF can prove the superiority of fusion on the basis of clinical or radiological outcome [35, 36]. In this study, we did not perform fusion in all cases, and two cases suffered implants failure; the implant failure rate (3.8%) was similar to other studies [37–39]. It was noteworthy that the 2 patients with implant failure were older than 60 years, which suggested that SSPF may not be the most proper method in patients with poor bone condition.

Several studies have reported good outcomes in thoracolumbar burst fracture patients treated by only SSPF without fusion and laminectomy [27, 40], while few cases in which SSPF and laminectomy are used without fusion have been reported. The necessity of laminectomy as a procedure for decompression in patients with neurological deficit is still in debate. The efficiency of laminectomy seems to be questionable, and it may destabilize the posterior column and increase kyphosis and fixation failure; however, it will remain controversial until evidence-based guideline is available. Since the use of rigid implants make the destabilization of vertebra is less of concern, laminectomy may be performed for decompression when indicated [10]. In this study, the use of SSPF and laminectomy without fusion could achieve significant canal clearance, steadily fixation, and satisfactory restoration of vertebral height and clinical outcome. Compared with long-segment instrumentation and fusion, this posterior-only approach has the advantages such as technically less demanding, relative safety, immediate pain relief by elimination of donor site pain, familiar anatomy for most surgeons, and less invasive. It was noted that there was significant decrease of canal encroachment and kyphotic angle, and the vertebral body height was restored significantly at last followup. As to clinical outcome, the neurological recovery was also acceptable. Only two patients developed screw breakage, but their kyphotic angle and neurological status did not deteriorate at last followup.

There is still no consensus about the relationship between canal encroachment and initial neurological status. Studies have revealed that the outcome of neurological injury depends on the severity and extent of damage to the neural elements at the time of injury, so the radiological static image of encroachment of the spinal canal after trauma is unable to represent the dynamic process and predict the neurological status [41–44], which is consistent with our study. However, there are still some studies which declare that the canal compromise is correlated with neurological deficit and advocate that the narrowing of spinal canal determines the neurological deficit in patients with thoracolumbar burst fracture [24, 45–47]. Besides, the Mohanty et al. [48] found that there is significant correlation between canal compromise and severity of neurologic deficit at T11 and T12 rather than L1 by subanalysis. Meves and Avanzi [46] concluded that a positive correlation was showed between narrowing of the spinal canal and neurological status in patients with incomplete neurological deficit, but such correlation was not present in patients with complete spinal cord injuries (Frankel A). In a word, the further researches and large-sample randomized controlled trials are still needed for determining whether canal compromise could be a predictor for initial neurological status of patients with thoracolumbar burst fracture.

In thoracolumbar burst fracture, the spinal canal narrowing is caused by the fracture bone fragments. Yan et al. [49] found that the strain in the spinal cord correlates well with the percent canal compromise. It has been suggested that the stenosis ratio of spinal canal is a risk factor related to the clinical results, and restoring a sufficient spinal canal may improve the chances of neurologic recovery [7–10, 20, 50–52]. It has been reported that for thoracolumbar burst fracture with incomplete paraplegia, SSPF could not achieve satisfactory reduction of retropulsed fragments by a posterior approach alone [40]. The direct decompression of spinal canal through posterior approach can be performed in posterolateral decompression. However, this approach has disadvantage of poor visibility of the retropulsed fragments. To prevent the SSPF failures, several techniques have been advocated such as transpedicular grafting, vertebroplasty, and balloon kyphoplasty. The usefulness of these techniques is still in doubt, and more attention should be paid to the potential dangerous situations such as the placement of graft and cement leakage [53–56]. The RRBF designed by the corresponding author makes the restoration easier, which could strengthen the anterior column, and then the removing of retropulsed bone fragments could be avoided. The repair of load-bearing capacity of the anterior column is widely considered to be crucial to prevent postoperative rekyphosing [25]. Moreover, repositioning rather than removing the bone fragments results in less tissue damage and time consuming. Another advantage is that the surgeon could estimate the extent of reduction by intraoperative fluoroscopy on the basis of reduction crank of RRBF. The laminectomy can provide clear visibility and convenient operation for reduction of retropulsed bone fragments by using RRBF. This technique was conducted in vitro prior to in operation, and the results confirmed a satisfactory canal clearance, safeness, and convenience.

There are several limitations in this study, such as it was a retrospective study and mean followup was 25.4 months. The long-term effectiveness of this technique still needs to be evaluated. The small sample of patients precludes absolute conclusion with regard to the advantages of this technique. Although this technique seems to be a potential therapy for thoracolumbar burst fracture, it is still unknown whether this technique is superior to others because no control group was available in this study. The outcome of this technique was satisfactory in this study; however, it by no means indicated that all patients with thoracolumbar burst fracture were appropriate for this treatment. The number of patients with AO 3.3 thoracolumbar burst fracture was too small to assure that this technique was proper for severe and comminuted fracture. The implant failure reminds us to be cautious when SSPF was implemented in patients with osteoporosis. It is inadequate to conclude that this technique could be a first line treatment for thoracolumbar burst fracture. However, mastering this technique will allow surgeon to be more flexible in specific situation, for example, patients who are not ideal surgical candidates for anterior approach, long-segment instrumentation, fusion, and vertebroplasty.

## Figures and Tables

**Table 1:** The relationship between the level of initial neurological status with the extent of canal encroachment, the loss of vertebral body height, and the kyphotic angle, respectively.

Neurological status	*n*	Canal compromise (%)	Loss of vertebral body height (%)	kyphotic angle (degree)
A	11	52.27 ± 9.73	42.18 ± 8.36	15.27 ± 4.00
B	16	50.38 ± 8.65	38.06 ± 12.34	15.75 ± 6.87
C	16	52.88 ± 7.32	41.63 ± 8.73	19.69 ± 8.11
D	10	48.90 ± 6.77	38.90 ± 5.78	17.70 ± 6.83
Total	**53**	51.25 ± 8.09	40.15 ± 9.40	17.21 ± 6.87
		*P > 0.05	*P > 0.05	*P > 0.05

*Statistical results of analyzing the relation between three radiological index and neurological status preoperation.

**Table 2:** The neurological recovery of 53 patients with thoracolumbar burst fracture.

Neurological status at admission	Neurological status at last followup				
	A	B	C	D	E
A	6	3	2	0	0
B	0	1	6	8	1
C	0	0	0	12	4
D	0	0	0	1	9
